# Evaluating head and neck trauma in surfing: injuries and prevention in an olympic sport

**DOI:** 10.1007/s00701-026-06844-0

**Published:** 2026-03-25

**Authors:** Caleb Ratz, Parker Dhillon, Brian Fabian Saway, Mustafa Al-Batryni, Liz Iglesias, Alejandro Spiotta, Stephen Kalhorn

**Affiliations:** Department of Neurosurgery, Medical University of South Carolina, 96 Jonathan Lucas St #307, Charleston, SC 29425 USA

**Keywords:** Surfing, Sport epidemiology, TBI, Return to play guidance, Helmet usage and safety

## Abstract

**Background:**

Surfing's Olympic inclusion has spotlighted it as an internationally popular, high-impact sport with risks of neurosurgical-relevant trauma. However, research on these injuries remains sparse and heterogenous and no synthesis has mapped this literature. All athletic levels of surfers face unique biomechanical forces from wave impacts, board collisions, and seafloor contact, which results in a significant, yet under-quantified, neurotrauma prevalence. Despite parallels with other action sports, surfing-specific epidemiology, helmet efficacy, and standardized return-to-play guidelines remain underdeveloped, even within professional and collegiate athletic governing bodies like the World Surf League (WSL).

**Methods:**

A scoping review screening for head trauma in surfing was conducted and yielded 25 studies published between 1976 and 2025 encompassing 72,093 patients. Epidemiologic data, injury mechanisms, reported intracranial pathology, need for neurosurgical intervention, and protective equipment use was extracted and synthesized in Table 1*.*

**Results:**

Our analysis of literature indicates an under quantified risk of neurosurgical-relevant trauma. Injuries include concussions and lacerations as the predominant mechanism of injury, and severe outcomes like skull fractures and intracranial hemorrhages, reported at 0.8–1.9% of large ED cohorts, which occasionally require neurosurgical intervention and, rarely, result in mortality. Direct surfboard impact is the predominant injury mechanism, estimated in one dataset to account for up to 55% of emergency department presentations. Higher rates of head injury were observed among pediatric surfers and those able to perform advanced maneuvers. Helmet use was inconsistently reported, noted in 11/25 studies, and its efficacy largely unevaluated. A critical finding is the complete absence of validated return-to-play guidelines in the reviewed literature. The prevalence of surfing injury is substantially underreported due to methodological limitations in existing studies.

**Conclusion:**

This review underscores an urgent need for standardized injury surveillance, rigorous protective gear research, evidence-based concussion management frameworks, and heightened neurosurgical awareness of the risks inherent to this growing global sport.

## Introduction

The inclusion of surfing in the Tokyo and Paris Olympic Games marks a watershed moment in the sport’s global trajectory, elevating both its visibility and participation. Surfing now engages millions of people worldwide, many of them recreational or novice participants drawn by its cultural appeal and growing accessibility. This expansion has opened the door for a broader population to enter high-risk surf environments, including reef breaks and powerful waves previously navigated only by elite professionals.

To meet the demands of global audiences, professional tours like the World Surf League (WSL) increasingly stage competitions at “waves of consequence,” such as Pipeline in Hawai ‘i and Teahupo ‘o in Tahiti, where powerful surf and shallow reef bathymetry significantly amplifies the risk of severe injury to professional surfers [6]. With a push towards critical surfing viewership and judging criteria favoring aerial maneuvers, recreational surfers are more likely to attempt high-risk surfing. These factors, along with the rapid evolution of the sport, has outpaced efforts to systematically understand and mitigate its neurological risks. As participation grows and surfers of all experience levels push performance boundaries, they are inevitably exposed to an increased incidence of high-risk biomechanical mechanisms: forceful ocean surface impacts, collisions with the surfboard and seafloor, and chaotic underwater turbulence, all of which pose significant risk for head and spine trauma.

These forces constitute a growing, yet poorly characterized, burden of traumatic, neurosurgical-relevant, injury in surfing. Despite well-established trauma incidence in analogous action sports such as skiing, snowboarding, and American football, surfing-specific epidemiologic head and neck trauma data remains limited [39]. Incidence rates of concussions and related traumatic head injuries across recreational and professional populations have not been comprehensively defined in surfing literature. This gap is compounded by a lack of surf-specific biomechanical research and limited data on protective headgear, in which adoption is inconsistent due to performance concerns and cultural resistance [46].

This review addresses a critical gap by mapping the existing literature on neurosurgical relevant surfing injuries. For this analysis, neurosurgical-relevant injuries were defined as trauma involving the head, face, neck, or spine with potential neurological consequences. Although many craniofacial lacerations do not require neurosurgical intervention, they were included due to their high prevalence among surfers and relevance as a mechanism of head trauma in the surfing population. Given the heterogeneity and limited depth of available studies, our objective is not risk quantification but characterization of current evidence regarding epidemiology, biomechanical mechanisms, and protective measures such as helmet use. This scoping approach allows identification of key trends, knowledge gaps, and collaborative opportunities to inform future research, safety innovation, and the development of surf-specific injury prevention and concussion management frameworks.

Initially conceived as a systematic review focused on traumatic brain injury (TBI) in surfing, this study was reframed as a scoping review to more appropriately capture the breadth and heterogeneity of surf-related trauma. Early screening revealed substantial body of high-relevance literature describing concomitant cervical spine and facial injuries, supporting expansion of the review scope and methodology.

## Methods

### Literature search strategy

A scoping review was conducted following PRISMA-ScR guidelines, searching PubMed and Scopus in April 2025 [44]. Retrieved studies were imported into Rayyan to remove duplicates. The search strategy employed the Boolean operators “OR” and “AND” with the terms: (Surfing OR Surfboard) AND (Head trauma OR Cranial injury OR Cerebral insult OR Concussion OR Traumatic brain injury OR TBI).

### Study selection criteria

Criteria for inclusion and exclusion were established a priori. Studies were incorporated if they included a minimum of five patients diagnosed with surfing-related TBI, presented data on these patients detailing their clinical attributes, and were authored in English. Studies were excluded if they took the form of book chapters, conference abstracts, case reports, technical narratives, editorials, letters to editors, reviews, or those involving animal or cadaver subjects. Studies that profiled patients with surfing-associated TBI but omitted excessive specifics on clinical characteristics were not considered.

Because many studies reported TBI within broader cohorts of head, facial, or spinal trauma and did not separate these injuries, we extracted neurosurgical-relevant injuries to allow a more complete representation of the injuries reported in surfing populations, while preserving our original TBI-focused inclusion criteria.

Additionally, studies that evaluated ocean surfing-adjacent sports, such as “skurfboarding,” windsurfing, and kiteboarding, were excluded to maintain relevance to wave-propelled biomechanics and injury mechanisms pertinent to ocean-surfing. River surfing and bodyboarding cohorts were included in our dataset due to comparable wave dynamics and head impact pathways. Mixed aquatic sports data were included if ocean surfing specific data could be isolated.

**Fig. 1:**
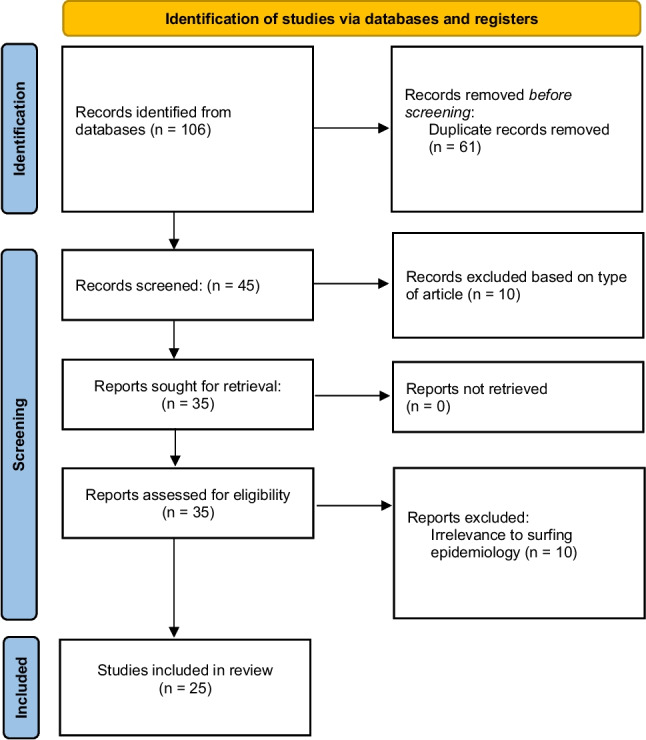
PRISMA-ScR Flowchart: PRISMA-ScR flow diagram for review, detailing initial database search numbers, records screened, and studies included

Two independent reviewers (P.D. and C.R.) screened titles and abstracts, followed by a full-text evaluation of potentially eligible studies. A third reviewer (B.S.) resolved discrepancies. Based on the previously defined criteria, pertinent publications were incorporated, with their bibliographies inspected for further relevant studies. Figure 1 outlines the final selection process.

### Data extraction

The data extraction process was initiated by reviewer M.A. and subsequently validated for accuracy by reviewers L.I. and C.R. Extracted data included study design characteristics, participant demographics, and key clinical variables such as injury type, mechanism, severity, neurosurgical interventions, and the use of protective equipment. In our study, the classification and weighting of evidence were guided by the 2011 Oxford Centre for Evidence-Based Medicine (OCEBM) Levels of Evidence as outlined by Howick et al. [18].

### Data charting and narrative synthesis

The primary outcome was aimed at characterizing the epidemiology of surfing-related neurosurgical-relevant trauma and the prevalent risk factors associated with these injuries. Due to the heterogeneity of the studies, the results were summated narratively. The risk of bias and certainty within the included studies was evaluated by three independent authors (M.A., P.D., and C.R.) using the JBI checklists. The level of evidence for each article was gauged according to the 2011 OCEBM guidelines [34].

## Results

In total, 25 studies, published between 1976 and 2025 and encompassing 72,093 patients, met inclusion criteria for this review (Table 1). These studies originated from a diverse range of countries, including the USA, Australia, Canada, Germany, the UK, Israel, New Zealand, and the Netherlands. The evidence was primarily Level IIb and III, consisting mainly of retrospective database analyses, cross-sectional surveys, and case series.

**Table 1:** Summary of current literature: Summaries of current literature on the sport of surfing and the growing epidemiological relevance to neurological interventions. Overview of the included studies

Study ID	Authors—Year	Study Design—Level of evidence	Country of Study	Sample size (n)	Age in Years (Range, Median or Mean)	Sex (Male n (%))	Severity of Injury (mild, moderate, severe)	Functional Outcomes	Neurosurgical intervention needed?	Laceration (Yes, No)	Skull Fracture (Yes, No)	Traumatic SAH (Yes, No)	Traumatic ICH (Yes, No)	Other associated skeletal fx? (Yes, No)	Associated spine injury? (Yes, No)	Mechanism of head Injury	Follow-up duration	Protective measures used
1	Kennedy M 1976	IIb	Australia	500	17.2 (14–25)	495(99%)	Mild	N/A	No	No	Yes 1	No	No	No	Yes 2	N/A	N/A	N/A
2	Allen RH 1977	III	USA	5	20 (8–38)	33 (94%)	Moderate/Sever	“Permanent Paralysis”	Yes	No	Yes 4	No	No	Yes 2	No	Struck by board	N/A	N/A
3	Lowdon BJ 1983	III	Australia	337	21.8 (10–49)	328(97%)	Moderate/Severe	“Time Lost Surfing”	N/A	Yes 81	Yes 4	No	No	No	No	N/A	N/A	N/A
4	Nathanson A 2002	IIb	USA	453	28.6	1214(90%)	Mild/Severe	“School/Work/Life missed due to hospitilization”	N/A	No	No	No	No	No	No	Direct contact injuries	N/A	Protective headgear (helmets)
5	Taylor DM 2004	III	Germany	12	28.2 ± (7.9)	14(90.2)	Severe	“Time off/chronic pain”	N/A	Yes 11	No	No	No	Yes 5	No	N/A	N/A	Protective headgear (helmets)
6	Hay CSM 2009	IIb	UK	71	27(11–66)	57(80%)	Mild	N/A	N/A	Yes 55	Yes 8	No	No	Yes 22	No	N/A	N/A	Protective headgear (helmets)
7	Woodacre T 2014	III	UK	81	28 (17–65)	N/A	N/A	N/A	No	No	No	No	No	No	No	Struck by board	N/A	Protective headgear (helmets)
8	Furness J 2015	III	Australia	152	35.8 (11–70)	138(91.3%)	Mild/Severe	“miss more than one day of work, school, or surfing”	No	No	No	No	No	No	No	Direct contact injuries	N/A	N/A
9	Swinney C 2015	III	USA	50	(18—34)	30(88%)	Full range	N/A	Yes	No	Yes 2	No	No	No	No	N/A	N/A	Protective headgear (helmets)
10	Klick C 2016	IIb	USA	470	27(19–37)	385(81.9%)	mild	closed head injuries—highest proportion of admissions (13.4%)	no	Yes 180	Yes 16	No	No	Yes11	No	N/A	N/A	Protective headgear (helmets)
11	Jubbal KT 2017	IIb	USA	93	37.8 (16–67)	37(90%)	severe	31 out of 93 patients (34%) suffered TBIs	Yes	No	No	No	No	No	Yes 34	N/A	N/A	N/A
12	Buzzacot P 2018	IIb	Australia	12,024	N/A	N/A	mild	90% managed in ED were treated and released	N/A	No	No	No	No	No	No	Struck by board	N/A	Protective headgear (helmets)
13	Kozminski BU 2019,	IIb	USA	34,337	25.6(4–74)	28,378(82.6%)	mild	N/A	N/A	No	No	No	No	No	No	Struck by board	N/A	N/A
14	Dean NA 2019	IIb	USA	7	N/A	4	mild	N/A	N/A	No	No	No	No	No	No	N/A	N/A	Protective headgear (helmets)
15	Lurie T 2020	IIb	USA	278	35–54	N/A	moderate	9% reported LOC	N/A	No	No	No	No	No	No	Struck by board	N/A	N/A
16	Furness J 2021	III	New Zealand	166	34.6 ± (11.9)	128(71.1%)	mild	N/A	N/A	No	No	No	No	No	No	Direct contact injuries	N/A	N/A
17	Szymski D 2021	III	Germany	52	28.0 ± (9.8)	N/A	mild	N/A	N/A	No	No	No	No	No	No	N/A	N/A	N/A
18	Yang SC 2021	IIb	USA	562	25 (2–78)	421 (74.9%)	Mild-moderate	N/A	N/A	Yes	Yes	No	No	Yes	No	N/A	N/A	N/A
19	Muhonen EG 2022	III	USA	19,729	N/A	12,567 (63.5%)	Moderate	N/A	N/A	Yes most common (46.1%)	No	No	No	Yes	Yes	Direct contact, hitting seabed, etc	N/A	N/A
20	Davidov B 2022	IIb	Israel	6	34 (20–50)	5 (83%)	Severe	2 blind with others having permanent sensory deficits	N/A	Yes 3	Yes 5	No	Yes 1	No	No	Struck by board	35.83 months	N/A
21	Quinn J 2022	III	Australia	884	30 (range: 2–77)	733(83%)	Mild	93.5% of cases were minor in nature and discharged from the ED	No	Yes 602 H/N lacerations	No	No	No	Yes	Yes	N/A	N/A	N/A
22	Hager M 2023	IIb	USA	297	N/A	243(82%)	Mild	N/A	N/A	No	No	No	Yes	No	No	N/A	N.A	Protective headgear (helmets)
23	Wende C 2023	III	Germany	128	36	N/A	Mild	52 (41%) needed to see a doctor	N/A	Yes	No	No	No	Yes	No	Contact with river bottom, board, side walls, maneuvers	N/A	38 (18%) reported using a helmet
24	Ward et al. 2024	IIb	Australia	40	21.7 ± 4.0	26 (65%)	Mild	N/A	no	No	No	No	No	No	No	N = 10 head vs water, n = 3 head vs surfboard = 2 head vs ocean floor, n = 3 rapid underwater	N/A	Helmet use reported n = 19, 14 positive, 23 negative reports
25	Snyder et al. 2025	IIb	USA	1359	3-81yrmean:29.2 ± 13.8	1088 (80.1%)	Mild	N/A	No	Yes	Yes	No	No	Yes	No	Board impact (NE = 48,360), Ocean floor (NE = 8690), Fin related (NE = 3610), Contact w surfer (NE = 2157)	N/A	N/A

*n* Sample size

*N/A* Not available/not applicable

*NE* National Estimate

Study Design/Evidence

**  • ***IIb, III* Oxford Centre for Evidence-Based Medicine evidence levels

Clinical Terms

**  • ***SAH* Subarachnoid hemorrhage

**  • ***ICH* Intracranial hemorrhage

**  • ***TBI* Traumatic brain injury

**  • ***LOC* Loss of consciousness

**  • ***Fx *Fracture

**  • ***ED* Emergency department

**  • ***H/N* Head/Neck

### Participant demographics

Sample sizes varied widely, from small case series, such as in Allen et al., to large national databases in Koziminski et al. [2, 7, 24, 25] Participant ages spanned from pediatric to geriatric, with a range of 2–81 years with a mean typically in the third decade of life [40, 49]. Across nearly all studies reporting sex distribution, the overwhelming majority of participants were male, frequently comprising 80% to 99% of cohorts. A recent ED database study by Hager et al. showed 82% of presentations were by males [16, 22, 36, 43].

### Injury types and epidemiologic trends

Significant study heterogeneity precluded calculating a pooled prevalence for injury types; however, a narrative synthesis reveals consistent trends. Lacerations to the head, face, and neck are the most common diagnosis. In a large survey of 1,348 surfers who reported 1,237 acute injuries, Nathanson et al. found lacerations to the head were the most common diagnosis, accounting for 42% (523 injuries). This is corroborated by ED database studies, where lacerations represented 40.7% of 2,072 surfing injuries and 46.1% of all craniofacial injuries in a cohort of over 54,000 cases [24, 35, 36]. In a 10-year audit of 2,680 ED presentations in Australia, craniofacial lacerations were the most common diagnosis at 37.9% [38]*.*

The reported prevalence of concussion and mild traumatic brain injuries (mTBIs) varies significantly, reflecting differences in reporting methods and growing awareness over time. Survey-based data show a wide range; in a U.S. web-based survey of 50 recreational surfers, Swinney et al. found that 13 participants (26%) self-reported having sustained a concussion, whereas in a larger survey of 1,348 participants, Nathanson et al. found concussions accounted for 6% (73 of 1,237) of all acute injuries [36, 42]. Data from a large USA based ED database show concussion rates between 2.7% and 7.5% of surfing-related injuries presenting for emergency care [16, 24, 35]. A critical trend was identified by Kozminski et al., who analyzed a similar national ED database of 34,337 weighted head injuries. They found that while the overall incidence of head injuries remained stable, the annual incidence and proportion of diagnosed mTBIs significantly increased between 2001 and 2016, rising from less than 5% to over 20% of all head injuries. A follow-up study by Snyder et al. found that from 2003 to 2022, overall head and neck injuries trended downwards, while concussion rates remained steady, suggesting improved diagnosis has established a "new normal" for reporting [40]. True incidence is likely much higher, as evidenced by Ward et al. which surveyed 40 elite Australian surfers, finding that while 13 surfers had a history of diagnosed concussion, the entire cohort reported hundreds of occurrences of post-wipeout concussive symptoms, suggesting many undiagnosed concussive events [46].

While less frequent overall, severe traumatic injuries such as fractures, intracranial hemorrhage, and spinal injury are consistently documented, especially in cohorts of hospitalized or trauma patients. Skull fracture rates were high in small case series of severe trauma (80–83%) but lower in larger ED cohorts (0.8–1.9%) [2, 7]. Hay et al. reported 4 skull fractures among 212 patients (1.9%) and Klick et al. identifying 16 cases among 2,072 patients in the E.D. (0.8%) [17, 24]. Intracranial hemorrhage (ICH or SAH) was also rare but present. Swinney et al. identified 2 SAH cases among 35 head-injured surfers (5.7%) [42]. Spinal injuries were also observed across both trauma-center and ED-based cohorts. In a study of 93 surfing-related cases at a Level 1 trauma center, Jubbal et al. found that 47 patients (51%) had injuries to the spine [20]. Additionally, Quinn et al. identified 33 cervical spine injuries among 2,680 ED presentations (1.2%) [38].

### Mechanisms of injury

Direct impact from the surfer's own surfboard is consistently identified as the leading mechanism of injury. Nathanson et al. reported that this mechanism accounted for 55% of 1,237 acute injuries, while Snyder et al. estimated it was the cause of 53% of all head and neck injuries in their 20-year analysis of United States ED data [31, 36, 40]. The second most common mechanism is impact with the ocean floor, including sand, rock, or reef, which was reported as the cause in 10% to 17% of injuries. In a study of 40 elite surfers, "contact versus the water surface" was identified as the primary mechanism for 10 diagnosed concussions, highlighting the role of deceleration forces [46].

### Neurological and neurosurgical outcomes

Severe neurological outcomes and the need for neurosurgical intervention, while not frequently noted, are documented in the studies reviewed. Permanent paralysis was reported in one case of bodysurfing-related spinal injury, and permanent blindness was documented in two of six patients with severe orbital trauma [2, 7]. The need for neurosurgical intervention was reported across multiple studies. In a cohort of 93 surfing-related trauma patients, 28 (30%) required surgical intervention, of which 19 procedures were for spinal injuries [20]. Allen et al. reported 1 of 35 hospitalized patients (3%) required a craniotomy, and Swinney et al. reported that 2 of 50 survey respondents (4%) required neurosurgical intervention for skull fractures or severe TBI [2, 42]. Critically, none of the included studies proposed or evaluated systematic Return-to-Play (RTP) guidelines for athletes recovering from TBI. Furthermore, no study directly evaluated long-term risks such as chronic traumatic encephalopathy (CTE).

### Protective equipment

Data on helmet use were inconsistently reported across the 25 studies, but where mentioned, usage rates are low [8, 31]. In a survey of 128 river surfers, 23 participants (18%) reported using a helmet [47]. Nathanson et al. found only 8% of 1,348 ocean surfers used a helmet. Among 40 elite surfers, 19 (47.5%) had tried a helmet, but cited mixed experiences regarding comfort and performance [36, 46]. No study systematically evaluated helmet effectiveness.

## Discussion

### Interpretation of findings

This review reframes surfing injuries as a significant neurosurgical concern and clearly highlights a spectrum of trauma that extends beyond superficial wounds. Our findings illustrate that the forces implicated in wipeouts can cause injuries that necessitate urgent intervention, highlighting a critical and under-quantified risk within the sport [2, 20, 42]. Although excluded from the primary analysis, surf-adjacent sports reinforce the observation that high-energy water board sports are not benign [5, 12, 29]. In kite surfing, Grunner et al. reported two cases of subarachnoid hemorrhage and one intracranial hemorrhage in a small, hospitalized cohort [12].

The predominance of direct surfboard impact as the primary injury mechanism is a critical finding, as it represents a potentially modifiable risk factor [24, 36]. While seafloor impacts more often result in severe trauma presentations, their lower frequency compared to board-related injuries suggests that interventions targeting equipment design could lead to significant impacts on injury reduction [35, 47].

Most included studies reflect recreational and emergency-department based populations, limiting formal risk stratification by experience level. However, certain subpopulations appear particularly vulnerable to surf-related neurotrauma. Notably, pediatric and adolescent surfers have been shown to experience higher concussion rates compared to adults. Hager et al. reported a pediatric incidence of 6.5% versus 3.2% in the adult cohort. Snyder et al. similarly reported that head trauma occurs more frequently in the pediatric population (8%) than the adult cohort (5%) among surfing-related emergency department presentations [16, 40]. Other research has consistently identified increased vulnerability among surfers that are able to engage in advanced maneuvers such as aerials, suggesting skill and age-dependent risk factors [10, 11, 22, 29, 30, 33].

Environmental conditions also shape injury patterns observed in surfing. Several studies describe injuries that occur during powerful wipeouts in shallow water where surfers may impact the reef, rocky seabeds, or sandbars, contributing to axial spinal loading, hyperextension, and severe traumatic injury [2, 20, 24, 36]. Wave height also influences injury patterns. Nathanson et al. reported that 47% of injuries occurred in overhead waves compared with 9% in double-overhead surf, likely reflecting the greater numbers of surfers present in moderate-to-large conditions, and the smaller population present in very large surf. In a subsequent analysis, the same group found that the risk of injury was more than doubled when surfing in large waves or over a hard seafloor [36, 37]. Additionally, crowding at popular surf breaks likely increases injury risk. Several studies note that collisions with surfboards or other surfers, events which are more likely in densely populated lineups, represent the most common mechanism of head injury [10, 17, 36, 46]. However, few studies stratify injures by surf break morphology, water depth, tide changes, wave biomechanics, or lineup density, leaving these environmental risk factors largely underquantified.

While rare, surfing mortality demands further characterization. A recent epidemiologic analysis from Australia identified 155 surfing-related deaths between 2004 and 2020. Drowning accounted for the majority of deaths (58.1%), while non-drowning cases accounted for 41.9%, including cardiac conditions identified in approximately one-third of cases (32.9%). Importantly, the authors note that traumatic injuries may be underrepresented as primary causes of death as trauma-related incapacitation may precipitate drowning events [27].

The lack of foundational data related to head trauma in surfing has direct implications for injury prevention and clinical care. No biomechanical force studies evaluating wipeout forces has been conducted to date. However, as evidenced by severe trauma presentations, surfboard and seafloor collisions likely produce forces comparable to analogous contact sports like football where the concussion threshold, based on both linear and rotational acceleration, is roughly 80–90 g [14]. Consequently, surfing lacks widely adopted concussion protocols and clinical return-to-play guidelines. Even among professionals, formalized TBI management is a recent development, and no clear framework exists for professionals or the much larger amateur and pediatric population.

The persistence of severe injuries, despite growing institutional advocacy for helmets, underscores the limitations of current preventive measures. Neurosurgeons in coastal regions or at surfing events must maintain a high index of suspicion for severe intracranial injuries like skull fractures and hemorrhages, even with mild initial presentations [2, 7, 12]. Furthermore, the increasing popularity of surfing in remote areas, with limited access to specialized neurotrauma care, presents a significant logistical challenge that needs addressing through improved local emergency response training and telemedicine support.

### Biomechanics of surfing injuries

Wipeouts subject surfers to a complex cascade of forces, including primary deceleration upon water impact, focused impacts from collisions with the board or seafloor, and secondary rotational forces from underwater turbulence [21, 40, 45]. The magnitude of energy transmitted during these events is governed largely by wave height, velocity, and the nature of the impact surface. These forces are further amplified by wave energy, which scales with height and period as defined by the wave energy flux equation [13].

Empirical data from contact sports suggest that the threshold for minor traumatic brain injury lies at forces exceeding 80 g, driven by both linear and rotational acceleration [14, 50]. Biomechanical studies in snowboarding, for example, have documented accidents on compacted snow and have been shown to generate head accelerations of up to 391 g (± 105 g), offering a potential comparison for seafloor impacts in surfing [3]. Compounded by secondary rotational forces underwater, direct impacts of a surfer on reef or sand may readily generate forces that meet or exceed concussion thresholds. Importantly, pediatric and novice surfers are especially susceptible to concussive forces even in smaller surf, as improperly executed falls and unintentional board strikes may still produce substantial linear and rotational forces [16]. In addition, the repetitive nature of both concussive and sub-concussive impacts in surfing raises concern for long-term neurodegenerative sequelae such as CTE, a phenomenon that remains entirely unstudied in this population.

The biomechanical forces leading to the high rate of spinal injuries seen in this review are distinct and critical. These injuries are often the result of direct axial loading (e.g., "piledriving" into the seafloor or a sandbar) or severe hyperflexion/hyperextension of the cervical spine during a forceful wipeout in shallow water [20, 39]. While TBI thresholds are measured in G-forces of acceleration, these spinal injuries are governed by compressive and shear forces that lead to vertebral fractures and ligamentous disruption [39]. This highlights a separate, equally dangerous injury-mechanism pathway that is also underquantified in the surfing population.

### Approximate global mTBI relevance

Extrapolating from the global surfing population, estimated to range from 23 to 35 million individuals, and applying an injury incidence rate of 3.5 injuries per 1,000 surfing days among recreational surfers, combined with average annual surfing frequency varying from 69 sessions in the Gulf region to 144 sessions in Hawaii, and incorporating a mild TBI prevalence of 16.1% among surf-related injuries, we estimate a global annual burden of mTBIs in surfers of approximately 894,000 to 2.84 million cases [26, 36, 40, 41]. Assuming roughly 50% of these cases will seek medical evaluation, we estimate the number of mild TBIs presenting for clinical care ranges from 447,000 to over 1.4 million annually worldwide. These findings reiterate a substantial global burden of head trauma in surfers.

### Olympic and Institutional Implications

With surfing's inclusion in the Olympic Games, governing bodies like the International Surfing Association (ISA) and the Olympic Committee face increasing pressure to address athlete safety. Consideration should be given to whether these organizations should regulate protective gear, including helmets, particularly in high-risk events or for specific age groups. The high-profile TBI sustained by Owen Wright at Pipeline in 2015, which influenced the WSL's adoption of a concussion protocol in 2022, illustrates the impact such incidents can have on policy and awareness [28]. So far, in the Championship Tour, the most prestigious competition with the most critical waves, athletes are required to have a concussion evaluation prior to the tour. However, with the “mid-season cut”, surfers can face negative consequences due to their injury [48]. Issues such as sponsorship withdrawal, grueling requalification after missing the cut, and strict ranking criteria based on participation lead to surfers playing through potentially serious injury and underreporting symptoms to maintain status and eligibility. Currently, no measures are employed by the Championship Tour and WSL to reduce these negative outcomes (i.e. rank protection due to medical clearance) [48].

### Protective equipment and barriers to adoption

Protective headgear presents a seemingly logical, yet deeply contentious, solution for mitigating head trauma in surfing [1, 4, 15, 46]. The data from this analysis clearly demonstrate a significant risk of trauma that helmets are designed to prevent, such as skull fractures and severe lacerations. However, the discussion surrounding their use is complicated by a lack of definitive data on concussion prevention, cultural and practical barriers to adoption, and the absence of analysis of the complex biomechanical forces at play in the sport.

Current surf helmets generally fall into two categories: hard-shell models (Gath and SIMBA helmets) and soft-shell designs (DMC Soft Surf helmet). The primary role and potential benefit of these current surf helmets, where hard-shell helmets are often certified to whitewater safety standards like EN1385, is the mitigation of high-energy, direct impacts to prevent catastrophic injuries like skull fractures [1, 46]. However, their efficacy in preventing concussions, a routine presentation in surfing, is largely unproven and no surfing-specific biomechanical studies analyzing head trauma have been done to date [46]. Concussions frequently occur from low-energy linear impacts or from the rotational and deceleration forces generated during a wipeout, which standard helmet designs may not sufficiently mitigate [23]. Given that a portion of this analysis identifies direct board impact as a primary injury mechanism, initial helmet design and testing should be tailored to these specific forces. Another point to consider is the comparison to sports such as amateur boxing, where helmets were controversially removed from elite male competition due to concerns that a larger head surface area could potentially increase rotational acceleration upon impact [39]. This raises important questions for surf helmet design, which must address both linear and rotational forces to be effective against concussion [46].

This lack of conclusive efficacy data creates a significant barrier to progress. Despite organizations like the WSL and ISA increasingly advocating for helmet use in high-risk scenarios, the lack of a definitive safety mandate perpetuates the status quo [48]. Compounding the data-driven challenges are deeply ingrained cultural barriers. Reported helmet use is consistently low, ranging from just 8% to 18% in various survey populations [36, 47]. This is partly due to the sport's "risk-taking culture," which often valorizes an individual’s skill, indicating natural consequences are simply a lack of ability. This frames protective gear as an impediment to performance or an admission of fear. Sociological analyses confirm that this type of voluntary risk-taking is closely linked to identity in extreme sports [32]. Elite surfers echo these cultural sentiments with practical complaints, citing that current helmets can impair balance, reduce spatial awareness, and create uncomfortable drag in the water [46].

With cultural stigma already hindering helmet adoption, reducing performance, through redesigned surfboard innovation, likely wouldn't be popular among the surfing community. Transferring helmet technology from other sports, such as slip-plane liners from white-water sports, could offer improved protection against rotational forces [9]. However, the limited research and inconsistent data on helmet use, despite an increasing participant population, underscore the need for more focused investigation [19].

A multi-faceted approach is required beyond simple advocacy. Robust, surf-specific biomechanical data is needed to guide next-generation helmet design, potentially adapting technologies like multi-directional impact protection systems (MIPS) from other sports. This includes quantifying the impact forces of board and reef collisions and, critically, the rotational forces experienced during wipeouts. Cultural change needs to be driven by education from data-equipped medical professionals and the advocacy of high-profile athletes who have experienced surfing-related trauma to shift perceptions. Until helmets are proven to prevent fractures and concussions and are designed in a way that aligns with the practical and cultural demands of the sport, their adoption will likely remain limited.

### Limitations of available studies

The current literature has critical deficiencies. Epidemiological data are limited by small sample sizes, retrospective designs, and heterogeneous reporting, leading to a substantial underestimation of injury incidence, further compounded by a lack of prospective, longitudinal investigations. Furthermore, variability in the application of standardized TBI severity assessment protocols impedes precise diagnostic categorization. Research on long-term outcomes, such as post-concussive syndrome (PCS) or CTE, remains conspicuously absent. Empirically validated RTP guidelines specific to surfing are similarly deficient. Of paramount importance, there is a paucity of investigations assessing the effectiveness of current surf helmets in preventing neurotrauma under impact paradigms. Comprehensive documentation regarding the modalities of neurosurgical intervention, patient outcomes, and logistical challenges in delivering specialized care within characteristic surfing environments is notably constrained.

### Future research directions

Future research should prioritize prospective studies with standardized data collection to overcome current limitations. Surf-specific laboratory and field-based biomechanical studies are needed to evaluate the efficacy of existing and emerging helmet designs in attenuating both linear and rotational forces under ecologically relevant impact conditions. Development of validated, sport-specific guidelines for TBI assessment, management, and return-to-play will require multidisciplinary collaboration among neurosurgeons, sports medicine specialists, and neurologists.

Longitudinal investigations are also essential to characterize the long-term neurological and psychological sequelae of both isolated and repetitive cranial trauma in surfing populations. Progress in this field will depend on coordinated partnerships among academic institutions, healthcare systems, governing organizations (including the WSL and ISA), and industry stakeholders, with controlled environments such as wave pools offering unique opportunities for biomechanical and equipment-efficacy testing.

## Conclusion

As a global Olympic sport, surfing carries a substantial, underestimated risk of surfing-related neurotrauma. Our analysis of 25 studies highlights a spectrum of head trauma, primarily from surfboard impact. The evidence base is weakened by inconsistent reporting and critical gaps in our understanding of TBI prevalence, long-term outcomes, and helmet efficacy. The absence of standardized, evidence-based return-to-play guidelines and the cultural barriers to helmet adoption further compound the risks faced by surfers at all levels. While current data are insufficient to definitively mandate helmet use across all surfing contexts due to limited efficacy studies and performance concerns, the documented severity of injuries strongly advocates for urgent, targeted research into helmet design and effectiveness, alongside enhanced education on TBI risks, and severe injury surveillance.

A paradigm shift towards proactive, evidence-driven injury prevention is needed, requiring an interdisciplinary collaboration among clinicians, engineers, researchers, governing bodies, and the surfing community. Prioritizing robust epidemiological surveillance, biomechanical research, development of effective and acceptable protective gear, and the establishment of clear clinical care pathways, including neurosurgical considerations, is paramount to safeguarding the neurological health of surfers worldwide as this physically demanding sport continues its global ascent.

## Data Availability

All data extracted for this scoping review are publicly available in the cited publications.
